# Immune and Nervous Systems Interaction in Endocrine Disruptors Toxicity: The Case of Atrazine

**DOI:** 10.3389/ftox.2021.649024

**Published:** 2021-03-10

**Authors:** Valentina Galbiati, Erica Buoso, Roberta d'Emmanuele di Villa Bianca, Rosanna Di Paola, Fabiana Morroni, Giuseppe Nocentini, Marco Racchi, Barbara Viviani, Emanuela Corsini

**Affiliations:** ^1^Università degli Studi di Milano, Milano, Italy; ^2^Department of Pharmacological and Biomolecular Sciences, Università degli Studi di Milano, Milan, Italy; ^3^Università degli Studi di Pavia, Pavia, Italy; ^4^Department of Drug Sciences, Università degli Studi di Pavia, Pavia, Italy; ^5^Department of Pharmacy, School of Medicine, University of Naples Federico II, Naples, Italy; ^6^Università degli Studi di Messina, Messina, Italy; ^7^Department of Chemical, Biological, Pharmaceutical and Environmental Sciences, Università degli Studi di Messina, Messina, Italy; ^8^Department of Pharmacy and Biotechnology, Alma Mater Studiorum - University of Bologna, Bologna, Italy; ^9^Università degli Studi di Perugia, Perugia, Italy; ^10^Department of Medicine and Surgery, Università degli Studi di Perugia, Perugia, Italy

**Keywords:** hormonally active compounds, immunotoxicity, neurotoxicity, steroid hormones, atrazine

## Abstract

Endocrine disruptors (ED) are natural and anthropogenic chemicals that can interfere with hormonal systems at different levels. As such, ED-induced alterations in hormone functions have been implicated in many diseases and pathological conditions, including adverse developmental, reproductive, neurological, cardiovascular, and immunological effects in mammals. The fact that ED may compete with several endogenous hormones for multiple receptors and pathways is not always fully considered. This results in a complex response that depends on the cellular context in terms of receptors and interacting proteins and, thus, may differ between tissues and circumstances. Microglia, neurons, and other immune cells are potential targets and still underappreciated actors in endocrine disruption. Due to the large scale of this topic, this review is not intended to provide a comprehensive review nor a systematic review of chemicals identified as endocrine disruptors. It focuses on the immune-neuro-endocrine network in ED toxicity and research gaps, using atrazine as an example to highlight this complexity and the interrelationship between the immune, endocrine, and nervous systems, and ED.

## Introduction

Over the past few decades, industrialized countries have faced a significant increase of diseases, including cancer, allergies, autoimmunity, and neurological disorders, such as cognitive and other neurodevelopmental deficiencies, that can be all linked directly or indirectly to alteration in the immune-neuroendocrine network (Manley et al., 2018). Environmental factors, including endocrine disruptors (ED), are believed to play an important role in such increased prevalence, with numbers that vary over time and from one region to another.

According to the European Commission, a substance is considered an endocrine disruptor if shows: “(a) an adverse effect in (an intact organism or its progeny)/(non-target organisms), which is a change in the morphology, physiology, growth, development, reproduction or life span of an organism, system or (sub)population that results in an impairment of functional capacity, an impairment of the capacity to compensate for additional stress or an increase in susceptibility to other influences; (b) an endocrine mode of action, i.e., it has the potential to alter the function(s) of the endocrine system; (c) the adverse effect is a consequence of the endocrine mode of action.” (Commission Regulation, 2018).

ED derive mainly from industrial and agricultural sources and include synthetic chemicals and their by-products (e.g., polychlorinated biphenyls, polybrominated biphenyls, dioxins), plasticizers (e.g., phthalates, bisphenol A), pesticides (e.g., atrazine, chlorpyrifos, dichlorodiphenyltrichloroethane, or DDT), fungicides (e.g., vinclozolin, organotins), and pharmaceutical agents (e.g., ethynyl estradiol; diethystilbestrol). Synthetic chemicals, heavy metals such as cadmium, lead, mercury, and arsenic have also been proposed as EDs (Kortenkamp, 2011). At the same time, we are also exposed to ED of natural origin mainly through diet, including phytoestrogens, such as genistein, daidzein, and coumestrol, or mycotoxins, such as zearalenone produced by numerous species of *Fusarium* (Demaegdt et al., 2016).

There is growing interest in the possible health threats posed by ED and their contribution to disease and disability with costs in the hundreds of billions of Euros per year (Trasande et al., 2015). ED are substances found in our environment, food, and consumer products that have effects on male and female reproduction, breast development and cancer, prostate cancer, cardiovascular, metabolism and obesity, and other disorders affecting the endocrine axis to mention some possible effects (Diamanti-Kandarakis et al., 2009; Kravchenko et al., 2015; Nahta et al., 2015; Lauretta et al., 2019; Zarean and Poursafa, 2019). Although there are many studies on this subject, it is outside the purpose of this article to provide a review of all epidemiological studies linking ED with adverse effects. Different mechanisms have been proposed to explain these associations but their complexity has prevented elucidation of the causal relationship between ED exposure and disease in the majority of cases. Additional *ad-hoc* experiments are needed to better explain this association. Atrazine nicely highlights this complexity and the interrelationship between immune, endocrine, nervous systems, and ED.

### The Immune-Neuroendocrine Network

Evidence is accumulating on the existence of multidirectional interactions among immune, nervous and endocrine systems (Manley et al., 2018). The endocrine system is critically important for immune and nervous system formation and functions and *vice versa*. Exogenous agents that disrupt the ability of the endocrine system to signal to the immune or the nervous system, can not only alter these networks but can subsequently be detrimental for the entire organism, resulting in an increased risk of both communicable (i.e., increase incidence of infections) and non-communicable diseases (i.e., allergy, autoimmunity, cancer, obesity, neurodegenerative disorders). Immune cells express hormone receptors, respond to signals from the endocrine system, and can produce several hormones, for example, enabling immune cells to promote anti-inflammatory effects in inflamed tissues more efficiently (Rubinow, 2018).

The immune-neuroendocrine network is a relatively new area of research in toxicology because most studies on ED primarily focused on reproductive and developmental toxicity. Investigations are needed to demonstrate the impact of ED exposure outside of the reproductive axis, and in particular on the immune and nervous system. We need to understand how the different systems interact with each other, to define whether they can be compromised or dysregulated with a subsequent loss, for example, of an effective tumor immunosurveillance network (Kravchenko et al., 2015), increased incidence of autoimmune disorders (Schooling and Zhao, 2015), or the development of neurodegenerative processes (Ferraz da Silva et al., 2018).

#### ED and the Immune System

Accumulated evidence indicates that the immune system can be a relevant target for hormonally active compounds (ED) with (anti)estrogenic and/or (anti)androgenic activities, especially following perinatal exposure, with important implications later in life for both communicable (i.e., increase incidence of infections, reduced response to vaccination) and non-communicable diseases (i.e., allergy, autoimmunity, cancer, obesity, neurodegenerative disorders, and all pathologies in which inappropriate inflammation is central to the progression). There is growing evidence indicating that ED can interfere with the immune system in humans and wildlife (Bansal et al., 2018). Effects are seen in both individuals exposed to ED and their offspring, suggesting that ED modulates the epigenetics of several cells of the body, including immune cells (Bansal et al., 2018). Studies have proved that ED, such as bisphenols, phthalates, triclosan, propanil, tetrachlorodibenzo-p-dioxin, diethylstilbestrol, tributyltin, and parabens can affect the development, functions, and lifespan of immune cells.

Of great concern are the possible consequences following *in utero* exposure, where developmental immunotoxicity encompasses xenobiotic-induced disruption of normal immune development resulting in enhanced susceptibility, or unique or more persistent effects on the immune system (Burns-Naas et al., 2008; Holsapple and O'Lone, 2012; Dietert, 2014). Developmental immunotoxicity is believed to have a significant role in later-life health and diseases (Dietert, 2014). Possible effects of chemical-induced developmental immunotoxicity are listed in Table 1.

**Table 1:** Developmental immunotoxicity.

**Target**	**Possible adverse effects**
Undifferentiated mesenchymal cells	Immune failure due to the lack of formation of hematopoietic stem cells
Cell migration	Thymic atrophy, impaired innate immunity, and inflammation
Immune organ development	Cancer, autoimmunity, and allergy
Functional development and maturation	Impaired Th1/Th2 balance, impaired memory with increased risk of infections, reduced response to vaccinations, increased risk of cancer, and allergy

Several animal and epidemiological studies have demonstrated that ED possesses both immunosuppressive and enhancing properties. On one side, they may increase susceptibility to infections and tumors, while on the other side, they may lead to inflammatory chronic diseases, allergy, asthma, and autoimmune disorders (Robinson and Miller, 2015; Bansal et al., 2018). Steroid hormones (androgens, estrogens, glucocorticoids, and progesterone) can act on immune cells by shifting the immune response toward either cell-mediated (e.g., androgens) or humoral immunity and inflammation (e.g., estrogens, progesterone) or anti-inflammatory (e.g., glucocorticoids). Surprisingly, only a few studies have evaluated the effects of ED on the differentiation of naïve T cells, resulting in the expansion of Th1, Th2, Th17, or regulatory T cells (Tregs) subsets. Very recently, Dong et al. (2020) demonstrated that BPA exposure during pregnancy and lactation could cause abnormal differentiation and function of Treg and Th17 cells in splenocytes of female offspring mice, with a decrease in the percentage of Treg cells and increase in Th17 cells, and a concomitant decrease in plasma TGF-β and increase IL-17. T cell differentiation is an epigenetic phenomenon able to permanently change the phenotype of T cells and determining the loss of immune homeostasis in the short- and long-term (Nocentini et al., 2018). Studies have focused mainly on Th1-Th2 balance, demonstrating that ED causes a shift toward Th1 or Th2 depending on the experimental conditions (Kato et al., 2013). No studies have evaluated the effect of ED on the differentiation of activated conventional CD4+ T cells in peripheral Tregs (pTregs) and the effect of ED on pTreg proliferation and survival. pTreg subsets (i.e., Tr1, Th3, and GITR single positive cell) are characterized by the expression at various levels of several markers peculiar to each subset, including GITR, TIGIT, CTLA-4, and FoxP3 (Petrillo et al., 2015; Roncarolo et al., 2018). They represent one of the main players in the control of the development of autoimmune/inflammatory diseases, but also in the inhibition of the immunosurveillance of tumors, thus favoring evasion of malignant cells and tumor growth (Nocentini et al., 2018; Cari et al., 2019).

#### ED and the Nervous System

Early-life exposure to ED is believed to be associated with neurobehavioral consequences and societal problems, greatly magnified late in life. In one scenario, the damage inflicted during early development remains silent until the compensatory capacity of the brain has eroded with aging and the latent damage emerges as functional impairment (Weiss, 2011). In large part, these effects appear to be linked to the interference with estrogen signaling and anti-androgenic properties.

Because of the emphasis largely focused on developmental outcomes, little is known on how ED might alter functions in the aging brain. The fact that endogenous estrogens, androgens, and thyroid hormones, all decline with aging, and that all three endocrine systems are vulnerable to ED should generate more recognition of their role in the aging brain (Weiss, 2011). Epidemiological reports and animal experiments have indicated a correlation between ED exposure, cognitive and behavioral alteration (Masuo and Ishido, 2011). The mechanisms underlying the effects of ED on the central nervous system (CNS) remain to be clarified.

The hippocampus is a brain area highly sensitive to hormonal action. Its position alongside the choroid plexus and immediately adjacent to the cerebral ventricles together with the expression of functional receptors for different hormones suggest that many blood and cerebral spinal fluid ligands can gain access to binding sites prominently in the hippocampus. The hippocampus is also a sensitive area to the action of ED, and data suggest that ED impair hippocampal functional plasticity and remodeling at the glutamatergic spine, through the recruitment of glutamatergic receptors (Parent et al., 2011). Sex steroids and thyroid hormones play a crucial role in the development of the hippocampus, by influencing cellular proliferation, dendritic outgrowth, or synaptogenesis. By altering the endocrine system, ED may affect these processes (Parent et al., 2011; Heyer and Meredith, 2017). These alterations seem to be irreversible as they persist in adulthood resulting in functional consequences across the lifespan (Parent et al., 2011), and are believed to play a role in intellectual impairment, attentional deficits, increased impulsivity, just to name some (Heyer and Meredith, 2017).

#### Cross Talk Between the Immune-Neuro-Endocrine Systems

The close connection of the immune system with other systems and tissues puts it at risk from secondary effects that arise from primary effects in other systems (DeWitt and Patisaul, 2018). Immune-neuro-endocrine interactions are for example responsible for indirect immunotoxicity, meaning that chemicals directly interfering with the endocrine axis or the nervous system may indirectly affect immune cells and immunological responses through the release of soluble mediators or axonal contact. The release of cytokines, neurotransmitters, hormones are used to communicate among the immune-neuro-endocrine systems.

A clear example of this interaction is the immunosuppression associated with stress (Cain and Cidlowski, 2017). The main function of the hypothalamic-pituitary-adrenal axis is bidirectional regulatory communication between the CNS and the hormonal system. This network regulates the immune system, and immune-derived cytokines (e.g., IL-1β, IL-6, TNF-α) activate the hypothalamic-pituitary-adrenal axis, by stimulating the secretion of the corticotropin releasing hormone, which in turn stimulates the pituitary to release the adrenocorticotropic hormone. The adrenocorticotropic hormone moves from the pituitary to the adrenal glands, promoting the secretion of glucocorticoids, which profoundly affect the immune system. Cortisol in humans and corticosterone in rodents are potent immunosuppressive and anti-inflammatory compounds, which help to control the immune system, preventing the immune response from becoming too strong (Manley et al., 2018). Moreover, stress can induce the release of catecholamines (epinephrine, norepinephrine, and dopamine) that signals an immunosuppressive effect throughout β-adrenoreceptors on immune cells, causing oxidative stress and type 2 responses (Eiden, 2013).

Other factors that can affect the immune-neuro-endocrine network include exposure to pathogens, physical agents, like ionizing radiations, temperature, and ED, like polychlorinated biphenyls, phthalates. ED can affect the hypothalamic-pituitary-adrenal or gonadal axes, leading to hormonal alterations of communication between the immune and the nervous system (Manley et al., 2018). Although ED can directly influence the immune-neuro-endocrine network, what we are missing is the mechanistic connection of these interactions. While it is clear that exposure to ED can affect the endocrine, the immune and the nervous system, the role of the individual system with respect to overall toxicity is less clear and remains challenging to ascertain. In addition, the sensitivity of ED in the different systems, especially following *in utero* exposure, is still a matter of debate (which tend to focus on questions relating to which came first).

## Atrazine

Although the use of the herbicide atrazine (2-chloro-4-ethylamino-6-isopropylamino-1,3,5-triazine, Cas N°1912-24-9) has been banned in Europe for several years, human exposure to atrazine continues to be a problem due to groundwater and sediment contaminations because of its metabolites desethyl-atrazine, deisopropyl-atrazine and diaminochlorotriazine persist for years, with chlorine metabolites being classified as toxicologically equipotent to the parent compound (Nödler et al., 2013). Furthermore, although banned in Europe, atrazine is still largely used in the rest of the world for control of broadleaf and grassy weeds, being the second most widely used herbicide after glyphosate (Atwood and Paisley-Jones, 2017).

In animal models, exposure to atrazine has been associated with reproductive dysfunction, behavioral abnormalities (e.g., impairment of motor, cognitive and emotional functions), and immunotoxicity in adults exposed during embryogenesis (Eldridge et al., 1999; Rowe et al., 2006; Cooper et al., 2007; Lin et al., 2014). Epidemiologic studies have associated gestational exposure to atrazine with impaired fetal growth (Chevrier et al., 2011), low birth weight (Almberg et al., 2018), male genital abnormalities (Agopian et al., 2013; Winston et al., 2016), and alteration in the timing of puberty (Namulanda et al., 2017).

The herbicidal action is based upon irreversible inhibition of photosynthesis through the blocking of the electron transfer at the reducing site of chloroplast complex II on thylakoid membranes. As mammalian mitochondrial electron transfer chain complex I and III have similar quinone binding sites to chloroplast and bacteria, it has been hypothesized that atrazine might bind to these sites causing mitochondrial dysfunction and oxidative stress, which, may be relevant to the toxicity observed in mammals (Jestadi et al., 2014).

### Effects of Atrazine on the Hormonal System

Regulatory agencies classify atrazine as an evident ED. Reduction in androgen levels and induction of estrogen synthesis - demonstrated in fish, amphibians, reptiles, and mammals—represents a plausible and coherent mechanism that explains the effect of atrazine on male development, consistent across all vertebrate classes examined (Hayes et al., 2011; Pereira de Albuquerque et al., 2020).

Atrazine and its metabolites do not bind to estrogen or androgen receptors (Eldridge et al., 2008). Atrazine, instead, enhances aromatase activity and serum estrogen levels in rats (Sanderson et al., 2002). Possibly related to epigenetic effects (Gely-Pernot et al., 2015), atrazine increases aromatase activity by binding to and inhibiting cyclic nucleotide phosphodiesterase, resulting in elevated cAMP production, sufficient to stimulate prolactin release in pituitary cells and to alter steroidogenesis (Sanderson et al., 2002; Roberge et al., 2004; Kucka et al., 2012).

Atrazine has been reported to affect levels of steroid hormones, including the activation of the hypothalamic-pituitary-adrenal axis with increased glucocorticoid and progesterone levels by centrally activating the axis through the corticotropin-releasing hormone (CRH) receptor (Foradori et al., 2017), and decreased gonadotropin production and disruption of related androgen-mediated processes, especially in males. It has been speculated that atrazine can act on CRH terminals in a fashion similar to γ-aminobutyric acid (GABA) or through GABA, to directly elicit the release of CRH, thereby driving the secretion of adrenocorticotropic hormone from anterior pituitary corticotrophs (Foradori et al., 2017).

The effect on thyroid hormones is conflicting and inconclusive, with earlier studies showing effects on thyroid hormones, both increasing (Kornilovskaya et al., 1996) and decreasing levels (Porter et al., 1999), and a study that observed no changes (Rooney et al., 2003). A study analyzing the effect of chlorinated metabolites of atrazine in Wistar rats also concluded that no differences in thyroid hormone levels [total thyrotropin (T4), and total triiodothyronine (T3)] or thyroid histology compared to controls were observed (Stoker et al., 2002). More recently, it has been demonstrated that embryonic exposure to atrazine at a relevant environmental concentration in caimans leads to thyroid gland disruption long after exposure ends, especially in females.

### Atrazine and the Immune System

Animal studies indicate important gender, genetic background, time, and effect differences in the immunotoxic effects of atrazine: mice are more sensitive than rats, juvenile animals are more sensitive than adult animals, and males more sensitive than females (Galoppo et al., 2020).

Prenatal/lactational exposure to atrazine affects the function of young adult rodent immune systems in a sex-dependent manner. Atrazine leads to immunotoxicity (mainly immunosuppression), with long-lasting effects in offspring as evidenced by the immunosuppression observed at 6 months in female mice (Rowe et al., 2008). While immunosuppression has been observed in female Balb/c mice (Rowe et al., 2008), and in male Sprague-Dawley rats (Rooney et al., 2003), increased cell-mediated and humoral immune responses have been reported in male Balb/c mice (Rowe et al., 2006). As atrazine is not considered to be transferred to the offspring through milk, the identity of any changes(s) in milk composition remains elusive, including a possible role of prolactin (Rooney et al., 2003). Even though the mechanism of action is currently unknown, one promising hypothesis relates to the epigenetic changes occurring during *in utero* exposure, which may affect gene expression and immune functions in offspring.

Juvenile *in vivo* studies indicate gender, genetic background, and species differences in the immunotoxic effects of atrazine (Filipov et al., 2005; Zhao et al., 2013; Lee et al., 2016). Taken together, the majority of these studies suggest a reduction in T lymphocytes and a reduction in immune functions. In C57Bl/6 male mice (4 wk old) exposed to atrazine a reversible decrease in spleen and thymus weights, alteration in cellularity with increased CD8+, and decreased MHC class II and CD19+ cells were observed (Zhao et al., 2013). In the same study, a decrease in spleen and thymus weights with degenerative changes, decrease in the % of CD3+ and CD4+ cells, decrease in Concanavalin A (ConA)-induced T cell proliferation, decrease in serum IL-4 (no changes in IL-2, IFNγ, or TNFα), and decrease NK cell activity were observed in females (Zhao et al., 2013). Similarly, (Lee et al., 2016) demonstrated in juvenile 4-week-old ICR male mice that atrazine induced a significant decrease in the number of spleen CD3+ T lymphocytes, while CD19+ B lymphocytes and non-lymphoid cells were unaffected as IL-4 induced IgE production, suggesting that atrazine may mainly target T cells.

Foradori et al. (2017) showed in exposed adult Sprague Dawley rats that the HPA axis was activated after a single dose of atrazine in males only, with statistically significant increases in corticosterone, progesterone, aldosterone. The response leveled off over time, possibly due to increased metabolic clearance of atrazine, and was not evident by day 7, although a significant reduction in DHT was observed in the 25 mg/kg/day atrazine-treated group on day 28. In the same study, no significant effect of atrazine treatment on prolactin levels was observed. No effects were observed on spleen and thymus weights, antibody production, or NK cell activity after 28 days of treatment. On the contrary, in adult male Balb/c mice atrazine decreased cell-mediated immunity, humoral immunity, and non-specific immune function (Chen et al., 2013). Karrow et al. (2005) observed an increased number of splenic CD8+ T cells, cytotoxic T cells, and mixed leukocyte responses in adult female B6C3F1 mice, with dose-dependently reduced host resistance to B16F10 melanoma. Thymus and spleen weights, total spleen cell numbers, and fixed macrophage function were also reduced in exposed mice. Zhang et al. (2011) demonstrated *in vivo* in adult female Balb/c mice the induction of apoptosis in splenocytes. A dose-related increase in the % of apoptotic lymphocytes was correlated with increased Fas/FasL and active caspase 3 as a reasonable mechanism of atrazine-induced decrease in spleen weight and degenerative micromorphology (Zhang et al., 2011).

*In vitro* studies, using both human and murine leukocytes, demonstrated the immunosuppressive effects of atrazine, indicating the direct effect of atrazine on immune cells as described below. As a possible scenario, starting from the hypothesized action at the mitochondrial level (Jestadi et al., 2014), atrazine may induce mitochondrial dysfunction and oxidative stress. Calcium increase may activate calcium/calmodulin-dependent protein kinase II enabling apoptosis through JNK-mediated Fas induction (Timmins et al., 2009), and oxidative stress may increase misfolded proteins leading to endoplasmic reticulum stress and apoptosis. This is supported by data published by Gao et al. (2016), demonstrating *in vivo* in Balb/c mice that atrazine induces the generation of reactive oxygen species (ROS) and increases intracellular Ca^2+^ in the spleen, increasing the level of advanced oxidation protein products, and causing a depletion of reduced glutathione in serum.

Atrazine exposure led to inhibition of cell growth and induction of apoptosis in human Jurkat T-cells (Lee et al., 2016). This study provides important clues on the molecular mechanism of action of atrazine. As previously demonstrated *in vivo* in Balb/c mice (Zhang et al., 2011), atrazine induces the activation of caspase-3 also in Jurkat T cells (Lee et al., 2016). In addition to caspase-3 activation, the cleavage of caspase-8 and PARP, with no release of cytochrome c from the mitochondria, was demonstrated, indicating the activation of the extrinsic apoptotic pathway as the main pathway of atrazine-induced lymphocyte apoptosis. They also demonstrated that atrazine activated the unfolded protein response (UPR) signaling pathway, as indicated by eIF2a phosphorylation and CHOP (C/EBP homologous protein or GADD153) induction, demonstrating that atrazine elicited an immunotoxic effect by endoplasmic reticulum stress-induced apoptosis in T-cells. In response to endoplasmic reticulum stress, cells induce an adaptive program called UPR, which facilitates folding by inducing the expression of chaperones; however, prolonged UPR can trigger apoptosis (Sano and Reed, 2013).

In addition to a direct effect on T cells, Pinchuk et al. (2007) demonstrated that atrazine also directly targets the dendritic cell line (murine JAWSII cells) maturation at not cytotoxic concentrations, decreasing MHC-I molecule, CD86, CD11b, and CD11c expressions, which is likely to contribute to the immune evasion observed *in vivo*. More than one mechanism is likely to be involved (see below), and the conflicting results observed may be explained by differences in experimental models, the dose used, biological samples investigated (cell lines, animal tissue, and human samples), parameters as well as differences in methodologies and experimental design.

### Atrazine and the Immune-Neuro-Endocrine Interaction

An attractive hypothesis to explain the toxicity of atrazine, linking together the immune-neuro-endocrine systems, is the effect on dopamine. It has been shown that dopaminergic signaling pathways have a central role in promoting homeostasis between the central nervous and the immune systems, and sex hormones can, by acting directly and indirectly on dopamine cell bodies, control dopamine synthesis via a multitude of mechanisms, including synthesis, release, turnover, and degradation (Sotomayor-Zarate et al., 2014; Vidal and Pacheco, 2020).

Dopamine receptors are broadly expressed on several immune cells with different effects. They can suppress or enhance both innate and acquired immune responses, depending on the dopamine concentration and the pattern of expression of dopamine receptors (Levite, 2016; Pinoli et al., 2017; Vidal and Pacheco, 2020). In addition, an autocrine/paracrine regulatory loop exists in lymphocytes, where dopamine is produced and released by immune cells, which act on receptors and influence the immune response (Thomas Broome et al., 2020).

Each regulatory circuit has positive and negative signals, and the signals will balance out physiologically, possibly under CNS control (Berczi and Katafuchi, 2012). Abnormalities of dopamine and dopamine signaling can lead to a range of neurodegenerative, psychiatric, and autoimmune disorders (Rangel-Barajas et al., 2015).

Exposure to atrazine has been reported to induce dopaminergic neurotoxicity, in both nigrostriatal and mesolimbic circuits, by disrupting vesicular storage and/or cellular uptake of dopamine, an effect believed to be due to the increase in cytosolic dopamine and subsequent generation of toxic oxidative metabolites (Coban and Filipov, 2007; Hossain and Filipov, 2008). The number of dopaminergic neurons in the substantia nigra pars compacta and ventral tegmental area of male juvenile C57BL/6 mice decreased after atrazine exposure (Coban and Filipov 2007). The decrease in dopamine-induced by atrazine has been linked to altered locomotor activity and coordination (Belloni et al., 2011). *In vitro* studies using PC12 cells treated with atrazine showed a dose-related reduction in intracellular dopamine (Das et al., 2003), via an alteration of the synthetic enzyme tyrosine hydroxylase and dopamine-β-hydroxylase.

As mentioned above, immune cells can not only express dopamine receptors, but lymphocytes and DC can also produce dopamine (Cosentino et al., 2007). Many effects of dopamine on immune cells have been described including activation of resting T effector cells (CD8+>>>CD4+), suppression of Treg, suppression of already activated T cells, modulation of Th1/Th2/Th17 differentiation (dopamine presents in dendritic cells polarizes vs. Th2, while depletion shift vs. Th1 differentiation), decrease ROS production, TNF-α production and neutrophil migration, decrease macrophage cytokine production, indicating its important immunomodulatory effect. The expression of dopamine receptors and functions are dynamic, context, and immune cells sensitive.

As a possible scenario, atrazine can directly inhibit dopamine synthesis and, indirectly, act as an estrogen-mimetic, decreasing dopamine level, which may block feedback regulation resulting in increased prolactin, alteration of immune cell activation, including T cell proliferation, DTH, and antibody response.

An interesting study, which could also offer additional clues on the immunotoxicity of atrazine, and link together the different effects, was published by Gely-Pernot et al. (2015) on the epigenetic effects of atrazine in the meiosis in male C57Bl/6 mice. Among the different genes modulated, very interesting is the increase in the orphan nuclear receptor NR5A2-binding sites. NR5A2 or liver homolog-1 is a member of the nuclear receptor family of intracellular transcription factors involved in the regulation of development, cholesterol transport, and steroidogenesis. NR5A2 may regulate several genes including CD95/FasL transcription [which may, together with increased ROS production, explain increased T cell apoptosis and altered effector T cell functions, (Schwaderer et al., 2017)], CYP19A1 or aromatase (which may explain decreased testosterone and increased 17β-estradiol), STAR, CYP2A1 (which may explain altered steroid hormones production), all contributing the understanding of the toxicity of atrazine. The ability of atrazine to activate NR5A was also demonstrated by Suzawa and Ingraham (2008), who showed that atrazine can activate NR5A *via* phosphorylation and increase cAMP and PI3K signaling. NR5A2 activation can be linked to oxidative stress, as demonstrated in liver treated with the NR5A2 agonist RJW101, in which superoxide dismutase 2 (Sod2) expression was increased and ROS production-induced by a high concentration of palmitate, was significantly reduced (Lee et al., 2017).

Dopamine and the hypothalamic-pituitary-adrenal axis have overlapping neurocircuitries, with bi-directional interaction between stress and the dopaminergic systems (Cabib and Puglisi-Allegra, 2012). A decrease in dopamine may result in excessive hypothalamic-pituitary-adrenal axis activation. The maturation of the hypothalamic-pituitary-adrenal axis responsivity may have a different developmental trajectory in atrazine-exposure compared to non-exposed animals during early life, which may result in alterations in physiological responsiveness and behavior (McEwen, 2008).

**Figure 1:**
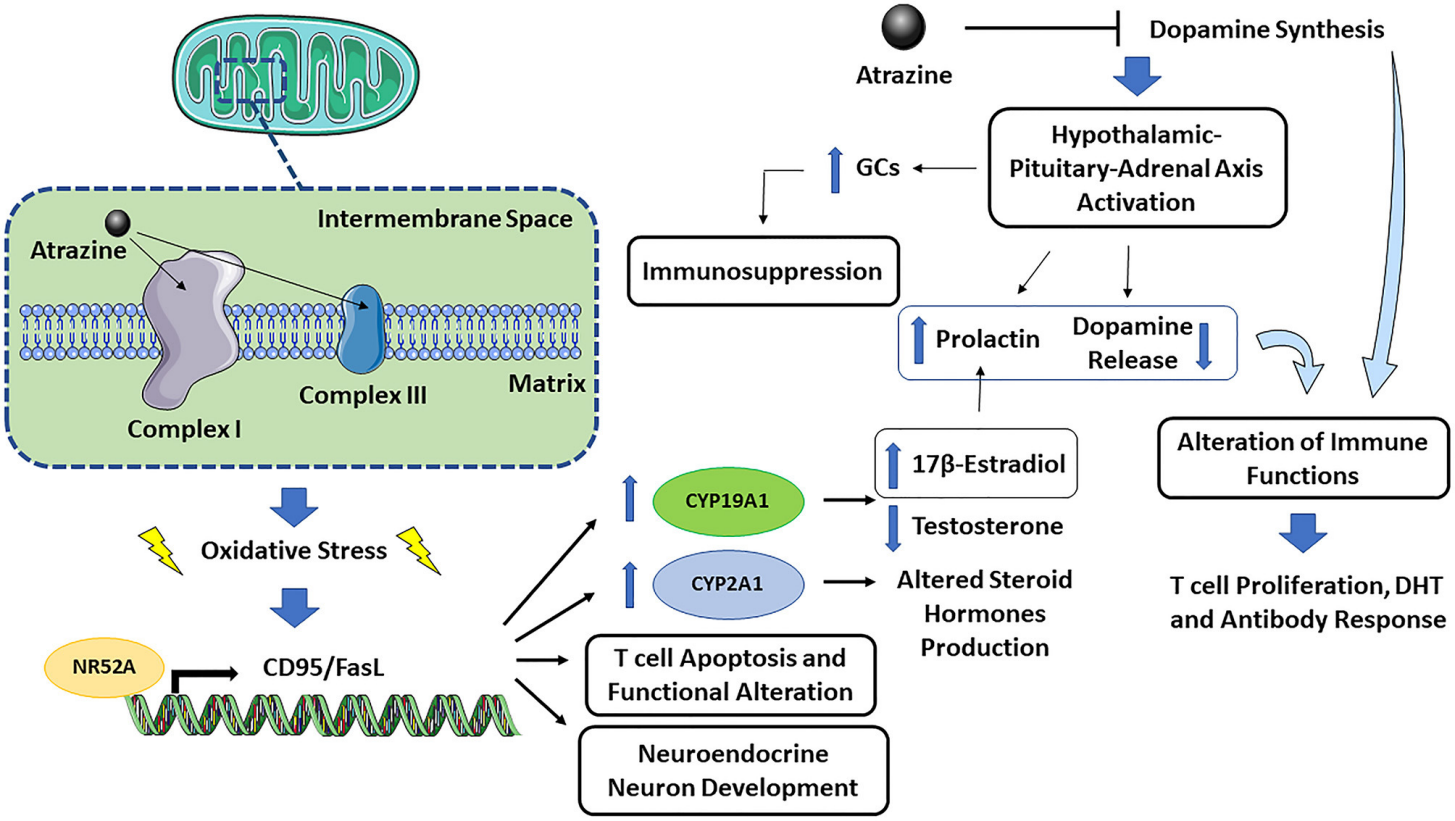
Graphical representation of atrazine neuro-endocrine and immune interaction.

The immune-neuro-endocrine systems are closely interconnected, and the effect of atrazine on dopamine synthesis is an example of such a network. Figure 1 presents a graphical representation of atrazine neuro-endocrine and immune interaction. The mechanisms contributing to atrazine immunotoxicity can be summarized as follows: (1) the binding of atrazine to mitochondrial electron transfer chain complex I and III may cause mitochondrial dysfunction and oxidative stress, followed by endoplasmic reticulum stress and apoptosis. As sex differences in the response to oxidative stress have been described, also at the cellular level, where female cells are generally more resistant to oxidative stress-induced cell death compared to males (Tower et al., 2020), one could expect an increased susceptibility to atrazine-induced immunotoxicity in males. (2) Oxidative stress may then activate, as a defense mechanism, the NR5A2 transcription factor, which in turn regulates CD95/FasL transcription. The latter, together with increased ROS production, explain increased T cell apoptosis and altered effector T cell functions (Schwaderer et al., 2017), CYP19A1 or aromatase induction (which may explain decreased testosterone and increased 17β-estradiol), CYP2A1 induction (which may explain altered steroid hormone production), and neuroendocrine neuron development (Alvarez-Bolado, 2019). (3) The ability of atrazine to inhibit dopamine synthesis may indirectly, through a CNS effect on hypothalamic-pituitary-adrenal axis activation or prolactin release, or directly as immune cells constitutively produce and respond to dopamine, affect immune cells. Dopamine is an immunomodulator, so if atrazine also reduced dopamine level in the peripheral system or the dopamine produced by immune cells themselves, this could result in adverse effects in the immune system (to be demonstrated). (4) The ability of atrazine to decrease dopamine synthesis may result in excessive hypothalamic-pituitary-adrenal axis activation, increased glucocorticoids, and immunosuppression. (5) Atrazine-induced estrogen production (via NR5A2-induced aromatase) might increase prolactin, and as feedback within the pituitary loop, resulting in reduced dopamine release. The increased prolactin and decreased dopamine will then alter immune cell activation, including T cell proliferation, DTH, and antibody response as observed in several studies [reviewed by Cosentino et al. (2007)]. (6) The conditions of exposure (dose, time, and the age at which exposure occurs) may differently affect the above-described mechanisms possibly explaining the different observed effects.

### Conclusions

Hormonally active substances have the potential to influence the immune and nervous systems, with developing systems being particularly vulnerable. On the other hand, chemical-induced toxicity can also result from mechanisms not necessarily associated with hormonal activity, as other nuclear receptors (i.e., AhR for TCDD) and intracellular signal transduction pathways (i.e., oxidative stress) have also different and important roles in regulating immune cell functions leading to immunotoxicity. As the activation of hormone receptors, i.e., ER/AR influences the expression of genes also regulated by other transcriptional events, including AhR, retinoic acid receptor, Toll like receptors and NF-κB activity, and vice versa, a complex interrelation and cross talk is likely to take place, which complicates understanding of the effects of hormonally active substances and the disentanglement of the contribution of the different pathways and receptors.

The fact that hormonally active substances may compete with several endogenous hormones for multiple receptors and pathways complicates the situation, resulting in a complex response that depends on the cellular context in terms of receptors and interacting proteins and, thus, may differ between tissues and circumstances. The example of atrazine highlights the complex interrelationship between the immune, endocrine, and nervous systems.

## Author Contributions

All authors listed have made a substantial, direct and intellectual contribution to the work, and approved it for publication.

## Conflict of Interest

The authors declare that the research was conducted in the absence of any commercial or financial relationships that could be construed as a potential conflict of interest.
